# Edge channels of broken-symmetry quantum Hall states in graphene visualized by atomic force microscopy

**DOI:** 10.1038/s41467-021-22886-7

**Published:** 2021-05-14

**Authors:** Sungmin Kim, Johannes Schwenk, Daniel Walkup, Yihang Zeng, Fereshte Ghahari, Son T. Le, Marlou R. Slot, Julian Berwanger, Steven R. Blankenship, Kenji Watanabe, Takashi Taniguchi, Franz J. Giessibl, Nikolai B. Zhitenev, Cory R. Dean, Joseph A. Stroscio

**Affiliations:** 1 Physical Measurement Laboratory, National Institute of Standards and Technology, Gaithersburg, MD USA; 2 Institute for Research in Electronics and Applied Physics, University of Maryland, College Park, MD USA; 3 Department of Physics, Columbia University, New York, NY USA; 4 Theiss Research, La Jolla, CA USA; 5 Department of Physics, Georgetown University, Washington, DC USA; 6 Institute of Experimental and Applied Physics, University of Regensburg, Regensburg, Germany; 7 Research Center for Functional Materials, National Institute for Materials Science, Tsukuba, Ibaraki, Japan; 8 International Center for Materials Nanoarchitectonics, National Institute for Materials Science, Tsukuba, Ibaraki, Japan

**Keywords:** Electronic properties and devices, Imaging techniques, Quantum Hall, Topological matter

## Abstract

The quantum Hall (QH) effect, a topologically non-trivial quantum phase, expanded the concept of topological order in physics bringing into focus the intimate relation between the “bulk” topology and the edge states. The QH effect in graphene is distinguished by its four-fold degenerate zero energy Landau level (zLL), where the symmetry is broken by electron interactions on top of lattice-scale potentials. However, the broken-symmetry edge states have eluded spatial measurements. In this article, we spatially map the quantum Hall broken-symmetry edge states comprising the graphene zLL at integer filling factors of ${{\nu }}={{0}},\pm {{1}}$ across the quantum Hall edge boundary using high-resolution atomic force microscopy (AFM) and show a gapped ground state proceeding from the bulk through to the QH edge boundary. Measurements of the chemical potential resolve the energies of the four-fold degenerate zLL as a function of magnetic field and show the interplay of the moiré superlattice potential of the graphene/boron nitride system and spin/valley symmetry-breaking effects in large magnetic fields.

## Abstract (web summary)

The broken-symmetry edge states that are the hallmark of the quantum Hall effect in graphene have eluded spatial measurements. Here, the authors spatially map the quantum Hall broken-symmetry edge states using atomic force microscopy and show a gapped ground state proceeding from the bulk through to the quantum Hall edge boundary.

## Introduction

Nontrivial topology is often related to electronic systems with highly degenerate ground states^1^, the most famous recent example being twisted bilayer graphene displaying the enormous richness of physical phenomena^2–4^. The zero-energy Landau level in graphene is another well-known example of a highly degenerate electronic state. The nontrivial “bulk” topology results in specific topologically protected edge states that have been studied so far with limited success.

The integer QH effect occurs when a two-dimensional (2D) electron system is subjected to a perpendicular magnetic field^5–7^. The metrological precision of the Hall conductance is understood in terms of the topological invariant of the Chern number associated with the Berry connection^8–11^. The precise quantization of the Hall conductance is related to the absence of backscattering in topologically protected chiral one-dimensional edge states with opposite momentum directions at the device boundaries. Imaging of QH edge states has been challenging due to their limited spatial extent and their location at the boundaries of the quantum Hall system. A number of notable attempts include: scanning gate microscopy^12^, scanning single-electron transistor (SET) measurements^13^, scanning force microscopy^14,15^, scanning charge accumulation^16^, and scanning microwave impedance microscopy^17^. More recent intriguing progress in imaging quantum Hall edge states has been made using SQUID-on-tip measurements of graphene^18^, however, the authors were not successful in imaging any broken-symmetry states inside the graphene zLL as the technique is limited to a moderate magnetic field range.

The graphene Landau level structure is determined by a combination of the Dirac-like linear energy-momentum dispersion and the π-Berry phase associated with the Dirac point resulting in Landau energies ${E}_{N}=\pm\sqrt{2e{{\hslash }}{v}_{F}^{2}{B|N|}}$, where *e* is the elementary charge, ${{\hslash }}$ is Planck’s constant divided by 2π, ${v}_{F}$ is the Fermi velocity, *B* is the magnetic field, and $N=0,\pm1,\ldots$ is the Landau level index, resulting in a non-uniform Landau level spacing^19,20^. The zLL in graphene, with orbital index $N=0$, comprises a set of fourfold degenerate Landau levels that are fixed at the Dirac point in the absence of SU(4) symmetry-breaking effects. The nature of the zLL state has been one of intense interest within the framework of quantum Hall ferromagnetism with many competing ground states^21–27^. The richness of physics in this regime is dominated by the interplay between the Zeeman energy against the sublattice anisotropy of Coulomb interactions which lift the degeneracy of the SU(4) multiplet. Indeed, extensive theoretical studies of the ground state of the zLL have shown the existence of many competing phases with distinct symmetry-breaking properties^21–27^. Among these phases are the ferromagnet (F) state and the antiferromagnet (AF) state, where the latter may form a canted antiferromagnetic (CAF) state^24^. Other possible phases include a charge density wave (CDW) and a Kekulé state (KD) (see Fig. 18 in ref. ^24^ for a phase diagram of the zLL). The delicate balance between various competing interactions can be impacted by multiple factors. For example, transitions between different ground states may be induced by changing the contribution of the Zeeman energy by tilting the magnetic field with respect to the graphene sheet^26^, and through other microscopic variables which break sublattice symmetry, the prominent example being the moiré-induced superlattice^27^.

The various ground states of zLL are predicted to have qualitatively different edge dispersions and excitations, which might or might not be revealed in a conventional transport experiment. For example, near the quantum Hall sample boundary, the isospin states may disperse into positively dispersing (electron-like) and negatively dispersing (hole-like) states, leading to gapped or gapless edge modes, depending on specific ground-state symmetries^21,26,28,29^. The edge dispersion of the CAF ground state was predicted to change from gapped to gapless as a function of tilted magnetic field^28^. Recent experimental observations of a metal–insulator transition observed in transport measurements as a function of tilted magnetic field^26^ have been interpreted as the evidence of the CAF ground state in accordance with theoretical predictions^28^. More recent theory^29^, however, has shown that the metal–insulator transition observed in transport measurements cannot be used as an unambiguous identifier of the zLL ground state in graphene. More complex behavior is expected as the ground-state order parameter changes in the proximity of the quantum Hall boundary^29^. Imaging the spatial properties of these broken-symmetry states can thus shed light on revealing the competing interactions and make a direct connection with theoretical models.

In this article, we determine the energies and the spatial dispersion of the zLL in graphene with AFM Kelvin probe measurements at arbitrary filling factors. The degeneracy of the zLL observed in AFM measurements is lifted in a magnetic field with the zLL split into four sublevels. The energy splitting of the four sublevels is much larger than the Zeeman energy, indicating interaction-dominated physics. The spatial dispersions of the states at $\nu =0,\pm 1$ are measured demonstrating completely gapped spectra as the states progress from the bulk to edge boundary. We discuss these findings in terms of recent theoretical developments of the graphene ground-state properties.

## Results

**Fig. 1:**
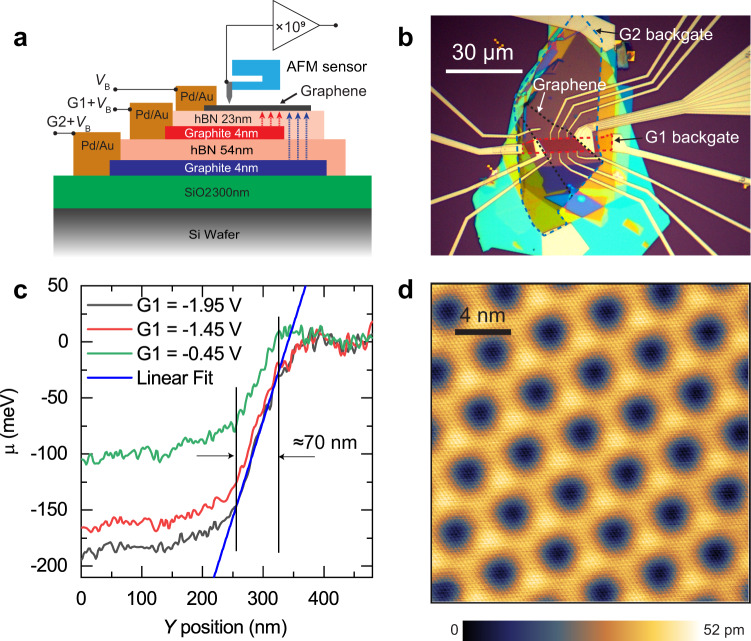
Graphene quantum Hall device structure. **a** Cross-sectional schematic of the layered structure of the graphene quantum Hall device. Two graphite back gates define the Hall bar: a global graphite back gate G2 (blue) and a local graphite back gate G1 (red). Pd/Au contacts are used to apply the sample bias ${V}_{\text{B}}$ to the graphene layer. **b** Optical image of the graphene device. The graphene sheet is indicated by the dashed black contour. The region controlled by the local gate G1 is shown by the dashed red line, while the region controlled by the global gate G2 is shown by the dashed blue line. Part of the fan “runway” used to guide the tip to the graphene is seen on the right side of the image. **c** Line traces from a Kelvin probe map at $B=0\,\text{T}$ at the local gate potentials indicated, showing the width and sharpness of the potential boundary due to the use of the graphite back gates in close proximity to the graphene layer. A linear fit to the trace at G1 = −1.95 V (black) over the region bounded by the vertical lines yields a slope of (1.72 ± 0.02) meV/nm, where the uncertainty is one standard deviation from the linear least-square fit. **d** STM topography of the graphene surface. Dark and bright spots represent the moiré superlattice formed by the graphene sheet and the hBN underlayer. The atomically resolved graphene lattice is visible as the fine mesh in the whole area. Topography is obtained at *V*_B_ = −100 mV and a tunneling current of 300 pA.

To image the QH edge states for this study, we chose to use a dual-gated graphene device where the Hall bar boundary is defined by a lateral junction controlled by two independent back gates (Fig. 1)^30–32^. The major advantage of this approach is the atom-scale cleanliness and precision of the boundary that is free of defects and contamination with the graphene lattice perfectly continuous across quantum Hall edge interface, as opposed to a physical boundary of a graphene sheet-shaped using harsh treatments, such as reactive ion etching. A similar device was used in imaging the edge states by SQUID-on-tip measurements of graphene^18^. Moreover, the area on one “external” side of the boundary can be tuned to an “electronic” insulator by setting the carrier density to zero (filling factor $\nu =0$) corresponding to an insulating state of graphene at high magnetic fields^33^. Such definition of boundaries of graphene devices has led to superior quantum Hall signatures in various geometries^34–36^.

**Fig. 2:**
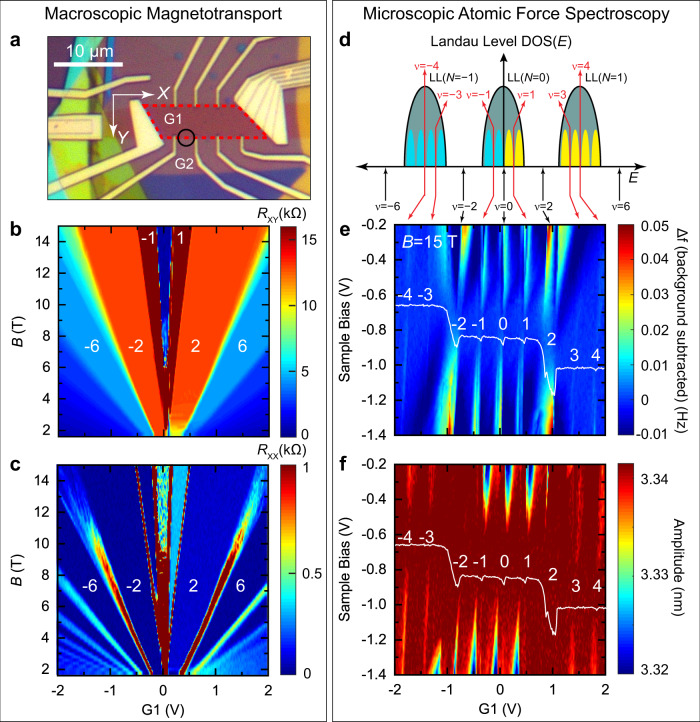
Correlation of graphene broken isospin states in macroscopic vs. microscopic measurements. **a** Optical micrograph of the graphene quantum Hall device. The Hall bar edges are defined by a local graphite back gate, G1, underlying the area outlined in the red dashed line, and a global graphite back gate, G2, under the entire Hall bar device (see “Methods” for further details). The boundary between G1 and G2 defines the quantum Hall edge boundary along the red dashed line. The black circle shows the location for spatial maps across the boundary shown in Figs. 4–6. **b**, **c** Magnetotransport measurements of (**b**) the Hall resistance, *R*_XY_, and (**c**) the longitudinal resistance *R*_XX_. Filling factors $\nu$ are indicated in white numerals. In both measurements, broken-symmetry states in the zeroth Landau level are observed at $\nu =\pm 1$. **d** Schematic of the graphene Landau level density of states indicating the fourfold degeneracy due to valley and spin inside each main Landau level. **e**, **f** Microscopic atomic force spectroscopy measurements revealing the broken-symmetry states in (**e**) AFM frequency shift measurements and (**f**) simultaneously obtained oscillation amplitude signal with constant excitation of 520 mV as a function of sample bias and local gate at *B* = 15 T. A smooth background was subtracted from the data in (**e**) to enhance the contrast of the broken-symmetry states (see Supplementary Fig. 2 and “Methods”). The white line indicates the zero-contact potential difference (i.e., chemical potential) obtained from a parabolic fit to the frequency shift data vs. sample bias (Supplementary Fig. 3). The white numerals indicate the filling factor. All measurements were made at $T=10\,\text{mK}$.

Figure 1a shows the schematic of the layered structure of the device and Fig. 1b is an optical image of the dual-gated device. The overlapped area determined by the graphene boundary (black dotted line) and local gate G1 boundary (red dashed line) in Fig. 1b defines the interior carrier density (area enclosed by the red dashed trapezoid in Fig. 2a). The second graphite gate G2 defines the exterior density. In most of the following measurements, the outside density is set to zero creating an insulating state at high magnetic fields, hence, defining the Hall bar geometry. The potential profile defined by these two back gates is quite sharp due to the thinness of the hBN gate insulator layers. The potential step is ≈70 nm in width as seen from the chemical potential measurements across the boundary in Fig. 1c. This characteristic scale is a few magnetic lengths at 5 T, providing sufficiently strong confinement for the quantum Hall edge channels.

The alignment of the graphene sheet with the underlying hBN dielectric is an important fabrication parameter often impacting the physical properties. In the case of this device, the graphene sheet is rotated relative to the hBN by about 3.1°, as determined from the moiré superlattice observed in STM topography measurements (Fig. 1d). This superlattice gives rise to an additional sublattice symmetry breaking potential generating a zero-field gap ${\triangle }_{\text{AB}}$. This potential can further affect the possible ground-state phase diagram adding a competing partially sublattice polarized (PSP) state in addition to the CDW and CAF states^27^. Recent transport measurements have indicated possible isospin phase transitions between these states as a function of magnetic field^27^.

Measurements of macroscopic and microscopic quantum Hall properties were made using an instrument that is capable of simultaneous magnetotransport, scanning tunneling microscopy, and AFM measurements on a given device at ultralow temperatures^37,38^. The instrument was operated at 10 mK for all measurements with a perpendicular magnetic field up to 15 T. Magnetotransport measurements of the Hall and longitudinal resistances are shown in Fig. 2b, c. Broken-symmetry states inside the zLL are seen at filling factors $\nu =0,\pm1$, marked by quantized plateaus in the Hall resistance (*R*_XY_). The same broken-symmetry states are observed in microscopic AFM measurements detected as changes in the frequency shift (averaged tip-sample force gradient) (Fig. 2e) and in the sensor oscillation amplitude (energy dissipation) (Fig. 2f) seen over a narrow density range around the integer filling factors, $\nu =0,\pm1,\pm2,\pm3,$ and $\pm4$. The AFM response to the symmetry-breaking states derives from the gapped nature of these states and their associated electronic incompressibility. The formation of an incompressible area under the tip apex leads to changes in the system capacitance and resistance, which alters the electrostatic average force gradient between tip and sample. This varies the sensor resonance frequency and hence enables the detection of the broken-symmetry states (see “Methods”). The contrast of the broken-symmetry states scales with the strength of the electrostatic field between the tip and the sample. It disappears at zero electrostatic field, at the sample bias which balances the work function difference between the probe tip and the sample, which is shown by the solid white line in Fig. 2e. The Landau levels are observed on the white line as plateaus with jumps or oscillations at the transitions between Landau levels at integer filling factors. The behavior will be investigated in more detail in chemical potential measurements using Kelvin probe force microscopy (KPFM) measurements, shown below.

### Kelvin probe force spectroscopy of graphene Landau levels

**Fig. 3:**
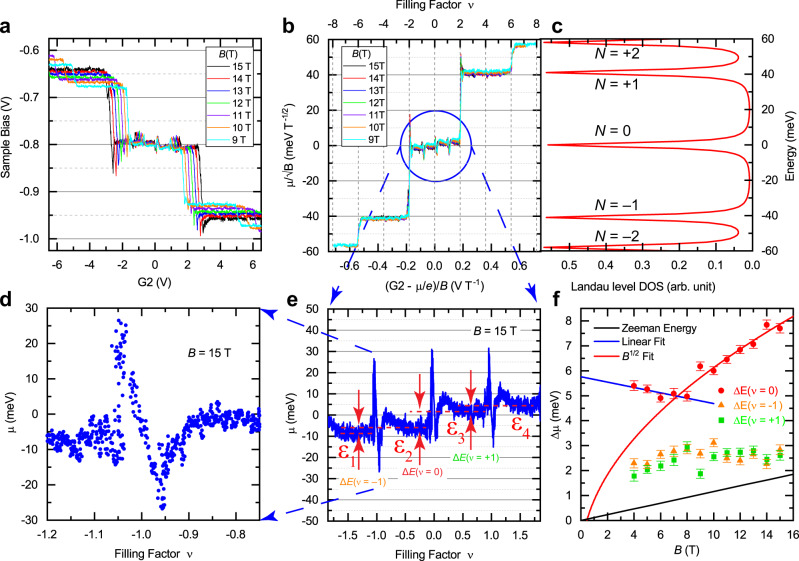
Resolving the energies of the four isospin components of the graphene zero Landau level with Kelvin probe spectroscopy. **a** Kelvin probe measurements varying the sample bias and simultaneously gates G1 and G2 for measurements made outside the Hall bar area. A staircase of plateaus shows various Landau levels occurring at different chemical potentials for various magnetic fields. **b** Chemical potential vs. filling factor given by the data in (**a**) collapsed onto a universal curve by scaling the sample bias by the graphene Landau level energy field dependence along the vertical axis, ${E}_{N}\propto {\!\,}^\surd B$, and by the ${B}^{-1}$ along the horizontal axis to give a density/filling factor axis. Each Landau level is observed by a plateau in the scaled chemical potential. Notice the zero Landau level at zero chemical potential consists of four separate small plateaus indicating the lifting of the fourfold degeneracy. **c** The Landau level density of states calculated using the expression in the main text with $B=1\,\text{T}$ and ${v}_{F}=1.13\times {10}^{6}\text{m}/\text{s}$ to fit the locations of the plateaus in (**b**). **d** Blow-up of the large up and down excursion in chemical potential at $\nu =-1$ and $B=15\,\text{T}$ from (**e**). **e** Blow-up of the chemical potential of the zeroth Landau level at $B=15\,\text{T}$ from (**b**) showing four individual chemical potential plateaus, labeled ${\varepsilon }_{i}$, separated by large up/down excursions at the incompressible filling factors, $\nu =0,\pm 1$. The red dashed lines indicate the differences in chemical potential $\triangle E=({\varepsilon }_{2}-{\varepsilon }_{1}),({\varepsilon }_{3}-{\varepsilon }_{2}),{\rm{and}}\,({\varepsilon }_{4}-{\varepsilon }_{3})$. **f** Energy differences extracted from the chemical potential plateaus in (**e**) for the $\nu =0,({\varepsilon }_{3}-{\varepsilon }_{2})$ (red circles) and $\nu =-1,({\varepsilon }_{2}-{\varepsilon }_{1}){\rm{and}}$ $\nu =+1,({\varepsilon }_{4}-{\varepsilon }_{3})$ (orange triangles and green squares) filling factors. The values are averaged chemical potential difference values from $\nu -0.75$ to $\nu -0.25$ of each integer $\nu$, and the error bars correspond to one standard deviation. The solid black line shows the Zeeman energy, $g{\mu }_{B}B$, with $g=2$. The solid red line is a fit for $\nu =0$ data values to $\sqrt{B}$ for values $\ge 8\,\text{T}$, and the blue line is a linear fit for *B* values $\le 8\,\text{T}$. AFM settings: 5.8 nm oscillation amplitude, $\triangle f=-450\,{\rm{mHz}}$, 5 Hz bias modulation, except a 1 Hz bias modulation was used for 4 T and 5 T data.

The frequency shift of the AFM qPlus probe, proportional to the average force gradient, shows an inverted parabolic profile as a function of applied electrostatic potential, characteristic of the electrostatic forces (Supplementary Fig. 3d). The vertex of the parabolic response occurs when the applied potential compensates the contact potential difference (CPD) between the probe and graphene and allows for measurements of the local chemical potential by KPFM^39,40^. Figure 3a displays KPFM measurements of the CPD as a function of the sample bias vs. back-gate potential G2 for different magnetic fields between 9 T and 15 T. These measurements were made outside the local gated area with the density of both areas kept the same by ramping G1 and G2 together with the appropriate scaling of gate voltages. A series of plateaus and transitions are observed at various back-gate potentials, depending on the magnetic field. Each plateau corresponds to the filling of a particular Landau level, whereas the transitions occur at the incompressible states when the Fermi level is being swept in the gaps between the Landau levels. The data in Fig. 3a show the characteristic graphene Landau level energy structure discussed above, as seen by scaling the sample bias by $\sqrt{B}$ and the gate potential by *B*, as shown in Fig. 3b. The correspondence to the graphene Landau level density of states is indicated by the lineup of the $N=0,\pm1,\pm2$ Landau levels in Fig. 3c with the plateaus in Fig. 3b.

On close examination of Fig. 3b, the $N=0$ Landau level plateau consists of four distinct smaller plateaus, as shown in the blow-up in Fig. 3e for $B=15\,{\rm{T}}$. The four plateaus, with the chemical potential labeled ${\varepsilon }_{i}$, indicate the complete lifting of the degeneracy of the $N=0$ Landau level. A large up and down, “N”-shaped excursion in the chemical potential is observed at the integer filling factors as transitions between the plateaus (see Fig. 3d and “Methods” section). Note that the excursion is characterized by very sharp upward jumps of the chemical potential over a small change of filling factor as the incompressible state is entered. The large excursion, on the order of ≈50 meV for $B=15\,{\rm{T}}$, is suggestive of interactions playing a strong role. Indeed, in previous measurements, an oscillating behavior of the chemical potential at integer filling factors was interpreted as exchange enhancement of the single-particle or broken-symmetry gaps due to Pauli exclusion^41^. Nevertheless, the sign of the excursion in our measurements, the peak followed by the dip, is opposite to that in previous measurements on the zeroth Landau level in graphene^42^, which requires further theoretical investigation. However, peak-dip patterns with negative compressibility have been seen in the phase transitions between correlated states in recent measurements of the flat-band system twisted bilayer graphene^43^.

Traditionally, the energies of the broken-symmetry states can be investigated by transport measurements only at integer filling factors assuming an activated behavior. These energies vary greatly between different devices, pointing to disorders contributing to the mobility gaps extracted from such activation measurements. In contrast to transport measurements, KPFM measures directly the local chemical potential (Fig. 3e) over a wide range of filling factors, both when the Fermi level is in the compressible state (plateau) and when it is in the incompressible state (between plateaus), complementing and expanding existing methods. These and other recent experiments^44^ will stimulate further theoretical analysis of partially filled Landau levels that has been lacking, partly because of a deficit of experimental data.

At integer filling factors, the bare symmetry-breaking potential is usually strongly enhanced by exchange and other correlations. The energy differences between the chemical potential plateaus, as indicated by the arrows in Fig. 3e at 15 T and shown in Fig. 3f for different magnetic fields, do reflect the strength of the lattice-scale symmetry-breaking potential but the degree of the enhancement is likely smaller than that at integer filling factors. The enhancement is still significant, as one sees that the energies are much larger than the Zeeman energy (solid black line), and the largest energy gap across $\nu =0$ $({\varepsilon }_{3}-{\varepsilon }_{2})$ reaches a value of ≈8 meV at 15 T. The energy gaps show distinct low- and high-field dependencies on the magnetic field. Starting with a plateau or even a slight decrease at fields below 8 T, the $\nu =0$ gap scales with $\sqrt{B}$ at high fields above 8 T. The scaling as $\sqrt{B}$ is consistent with electron interactions playing a dominant role. The $\nu =\pm1$ energy gaps, $({\varepsilon }_{2}-{\varepsilon }_{1})$ and $({\varepsilon }_{4}-{\varepsilon }_{3})$, show lower energies monotonically increasing to the highest field of 15 T. As noted above, the measured values of the $\nu =\pm1$ energy gaps are larger than the Zeeman energy. Interestingly, the $\nu =0$ and $\nu =\pm1$ energy gap dependencies can be interpreted as an avoided crossing at around 6–8 T, suggesting a possible change in the ground state. An isospin phase transition proposed in Ref. ^27^ occurs due to the influence of a moiré superlattice contribution. Indeed, the atomic resolution STM measurements of the device in Fig. 1a does show a moiré period of ≈4.36 nm corresponding to a misalignment of 3.1° of the graphene lattice relative to the hBN underlayer (Fig. 1d). An additional potential of the moiré superlattice causing the sublattice-symmetry breaking is generating a zero-field gap ${\triangle }_{\text{AB}}$. This alters the possible ground-state phase diagram to include a partial sublattice polarized (PSP) state in addition to the CDW and CAF states^27^. Magnetotransport measurements as a function of misalignment angle have shown that a zero-field gap scales with rotation angle^45^, and a gap value of 5–10 meV can be expected for the angle of 3.1°. This value is consistent with the behavior of $\triangle E(\nu =0)$ in Fig. 3f if the trend of the low-field regime is extrapolated to zero field (blue line). Respectively, the apparent avoided crossing seen in Fig. 3f at fields of 6–8 T could also be suggestive of a possible isospin transition between a CDW to AF phase at intermediate magnetic fields.

### The quantum Hall edge wedding cake-like potential profile

**Fig. 4:**
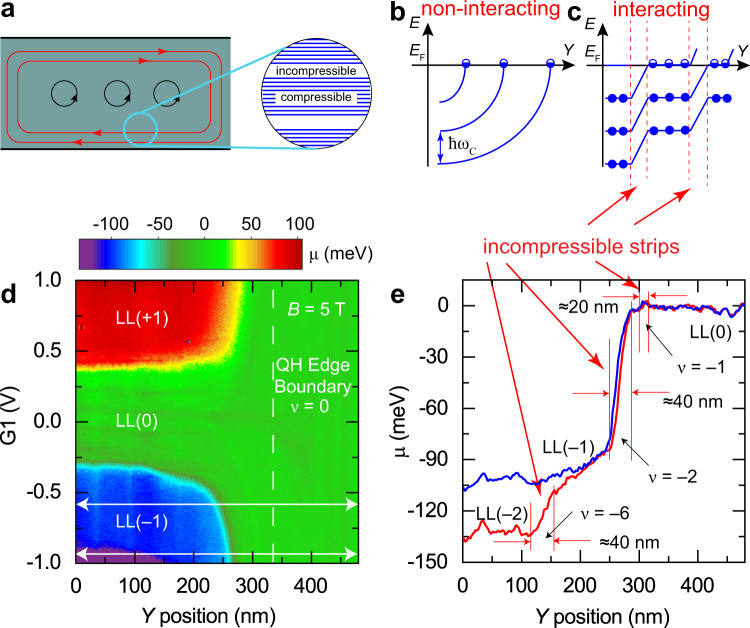
KPFM measurements of incompressible strips. **a** Schematic of bulk closed cyclotron orbits with cyclotron energy ${\hbar \omega }_{C}$ and edge quantum Hall states leading to compressible and incompressible strips at the device edge boundary. **b**, **c** Schematic of the bending of the Landau levels in a confining potential boundary (**b**) in a noninteraction picture and (**c**) in an interacting picture leading to a “wedding cake-like” series of plateaus in Landau levels near the boundary edge. A compressible strip is formed when a Landau level is at the Fermi level, separated by incompressible strips (red dashed lines) during Landau level transitions. **d** Kelvin probe map at $B=5\,\text{T}$ of the chemical potential as a function of *Y*-position across the quantum Hall boundary (indicated in the black circle in Fig. 2a) and local-gate potential. In the local-gate area, Landau levels from $N=-2$ [LL(−2)] to $N=+1$ [LL(+1)] are seen in the different colored plateaus. AFM settings: 2 nm oscillation amplitude, $\triangle f=-2\,{\rm{Hz}}$, and 20 mV sample bias modulation at 1.4 Hz. **e** Incompressible strips in the chemical potential are observed in the lines traces at ${\rm{G}}1=-0.9\,\text{V}\,\left({\rm{red}}\right)\,\text{and}\,{\rm{G}}1=-0.6\,\text{V}\,({\rm{blue}})$ (white horizontal lines in (**d**)) corresponding to filling factors $\nu =-6,-2,$ and $-1$. The transitions separate plateaus between Landau levels, LL(−2) to LL(−1), and LL(−1) to LL(0), confirming the “wedding cake-like” structure predicted in ref. ^46^ .

The edge states in the QH effect form a series of incompressible and compressible strips near the boundary edge (Fig. 4a). The strips originate from the Landau levels that are bent by the potential rise at the boundary and are pinned at the Fermi level (Fig. 4b). In a noninteracting picture, the levels intersect the Fermi-level forming one-dimensional edge states. In reality, interactions and screening reconstruct the potential into a series of plateaus forming a “wedding cake-like” structure (Fig. 4c)^46,47^. A compressible strip is formed when a partially filled Landau level is at the Fermi level, which is separated by incompressible strips as the next Landau level transitions to the Fermi level. The stepped potential profile near the boundary has been predicted theoretically for many years but has eluded measurement^46^. Using AFM Kelvin probe spectroscopy, a spatial visualization of the Landau levels from $N=-2$ [LL(-2)] to $N=+1$ [LL(+1)] is obtained as a function of *Y-*position across the QH edge boundary (indicated in the black circle in Fig. 2a) and local gate voltage G1, as shown in the Kelvin probe map at $B=5\,\text{T}$ in Fig. 4d. Figure 4e shows the extracted stepped potential across the QH edge boundary (see white arrows in Fig. 4d), transformed by screening from the bare potential in Fig. 1c. The profiles in Fig. 4e show the “wedding cake-like” steps with plateaus separated by sharp drops at the incompressible states, as theoretically predicted^46^. For the larger gate voltage (red line), we observe three incompressible strips corresponding to filling factor $\nu =-6,-2,{\rm{and}}-1$. The width of the $\nu =-6\,{\rm{and}}\,\nu =-2$, which occurs at the change of the Landau levels are on the order of ≈40 nm. The width of the $\nu =-1$ strip is much narrower, on the order of ≈20 nm. These are consistent with the length scale of the electrostatic potential of the graphite back gates defining the quantum Hall edge shown in Fig. 1c, as calculated below in more detail. A similar stepped profile of graphene Landau level energies has been recently measured in graphene quantum dots by tunneling spectroscopy^47^.

The staircase shape of the chemical potential near the QH boundary is caused by the screening dependence on the filling factor flattening out the potential in compressible states and displaying potential steps over incompressible strips^46,47^. The width of the incompressible strips, *a*, can be estimated using Eq. S25 in ref. ^47^,

1 $$a={\left(\frac{4(4\pi {\epsilon }_{0}){\epsilon }_{r}{\triangle E}_{\text{LL}}}{{\pi }^{2}{e}^{2}\frac{\partial n}{\partial y}}\right)}^{\frac{1}{2}}$$

Here ${\epsilon }_{0}$ is the vacuum permittivity, ${\epsilon }_{r}$ is the relative permittivity, *e* is the elementary charge, ${\triangle E}_{\text{LL}}$ is the energy gap between Landau levels, and $\partial n/\partial y$ is the density gradient at the strip position. For the density gradient, we can use the electric field profile across the boundary measured at zero field (Fig. 1c). Here, the boundary width is observed to be on the order of 70 nm, with a potential gradient of ≈1.7 meV/nm across the boundary. For the Dirac dispersion, the corresponding density gradient is $\approx {10}^{23}{\text{m}}^{-3}$ using ${v}_{\text{F}}=1.0\,\times {10}^{6}$ m/s, ${\epsilon }_{r}=5$. Substituting the density gradient and 80 meV for energy gap in Eq. (1) yields an incompressible strip width of 34 nm, in good agreement with the measured strips in Fig. 4.

### Spatial mapping of the broken-symmetry edge channels

**Fig. 5:**
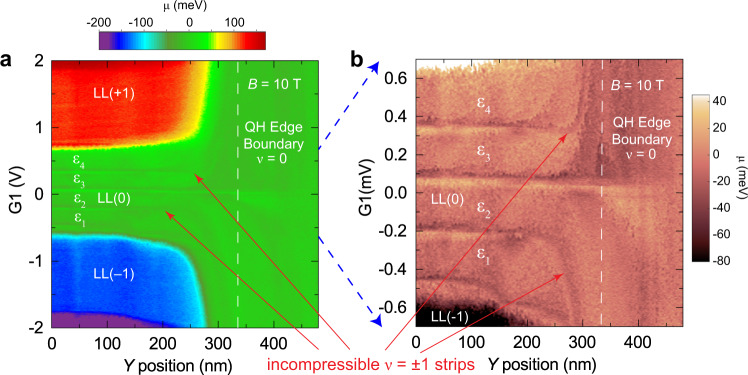
Spatial dispersion of graphene broken-symmetry edge states at the quantum Hall edge boundary. **a** Kelvin probe map at $B=10\,\text{T}$ of the chemical potential as a function of *Y*-position across the QH boundary and local gate potential. In the local gated area, Landau levels from $N=-2$ [LL($-$2)] to $N=+2$, [LL(+2)], are seen in the different colored plateaus. Incompressible signatures due to the large excursions in the chemical potential (see Fig. 3d) are observed inside the $N=0$ Landau level corresponding to filling factors $\nu =0,\pm 1$. **b** Higher-resolution Kelvin probe map of the $N=0$ Landau level showing that at the quantum Hall edge boundary the $\nu =\pm1$ channels disperse away from the $\nu =0$ center line. AFM settings: 2 nm oscillation amplitude, $\triangle f=-2\,{\rm{Hz}}$, 20 mV bias modulation.

**Fig. 6:**
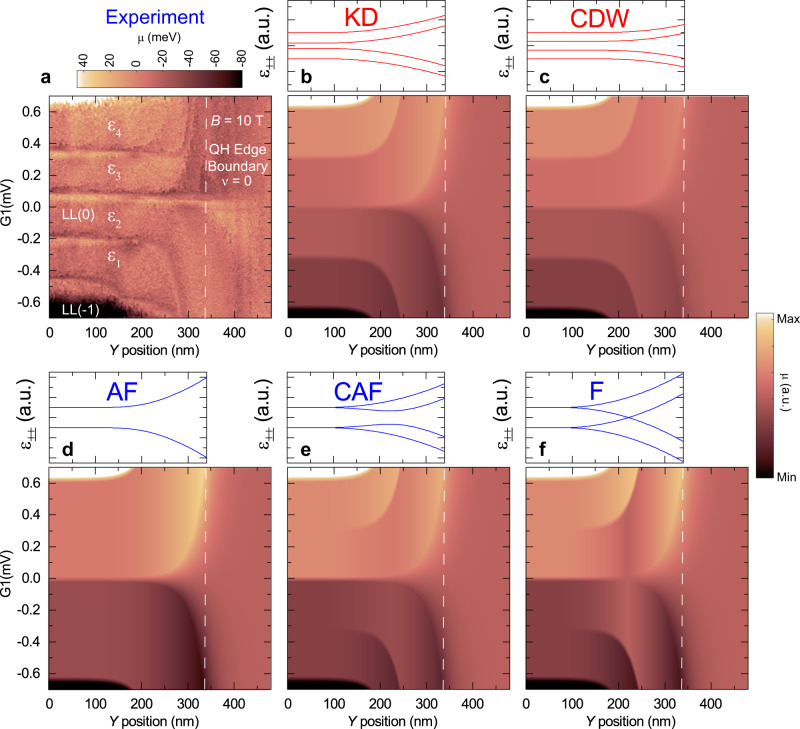
A comparison of the experimental Kelvin probe map of the graphene broken-symmetry edge states with different ground-state symmetries. **a** Kelvin probe map at $B=10\,\text{T}$ of the chemical potential as a function of *Y*-position across the QH boundary from Fig. 5b. **b**–**f** (top panels) Schematic dispersion of the four single-particle energy levels ε±± for different phases obtained from the analytic formulas in Table 1 of ref. ^29^. (bottom panels) Simulation of the Kelvin probe maps of the chemical potential vs. local gate and *Y*-position using the dispersions of the various ground-state symmetries in the top panels along with the measured gate response profile in the G1-*Y* plane obtained at $B=0\,\text{T}$, as shown in Fig. 1c.

The essential advantage of KPFM is that the measurement is compensating the CPD between tip and sample and thus minimizes gating effects in the graphene 2DEG. Figure 5 shows the KPFM measurements at $B=10\,\text{T}$ of the chemical potential across the QH edge boundary as a function of *Y*-position and local gate potential, G1. At a distance of about 300 nm from the boundary, the different chemical potential plateaus at the left edge of Fig. 5a correspond to the $N=0,\pm1,\pm2$ Landau levels, as in Fig. 3b. As the probe approaches the QH edge boundary, the electron (hole) states disperse to positive (negative) densities. The large excursions seen at integer filling factors in the chemical potential in Fig. 3e are useful as fingerprints for spatial mapping of the incompressible states and edge channels in the zeroth Landau level. Inside the $N=0$ Landau level, the $\nu =\pm1$ incompressible states and the ${\varepsilon }_{i}$ compressible channels are observed to follow a dispersion similar to the higher Landau levels, as seen in the higher-resolution measurement in Fig. 5b. Following the spatial dispersion to the boundary, we do not observe a crossing or oscillations in the energy of the edge states predicted for some ground-state phases of the zLL. Spatial mapping of the edge states in the *XY* plane shows the channels are uniform along the boundary edge, i.e., the *X* direction (Supplementary Fig. 4). The broken-symmetry states remain gapped starting in the bulk and proceeding to the boundary. The $\nu =-1$ incompressible edge channel is seen dispersing to below $\nu =0$ and $\nu =+1$ disperses above. For comparison, the edge dispersion and Kelvin probe simulated maps expected for the KD, CDW, AF, CAF, and F phases are shown in Fig. 6b–f, respectively^28,29^. We further compare the experimental dispersion with the predictions of these models in the “Discussion” section below.

## Discussion

Magnetotransport experiments have provided convincing evidence for the interaction-dominated nature of the partially filled zLL in graphene. In high-mobility samples, the observed large longitudinal resistance at $\nu =0$ indicates that both bulk and edge states are gapped^33^. Such states belong to the class of quantum Hall ferromagnets with broken symmetries in the spin-isospin space^23^. The key physical challenge is identifying how the symmetry is broken as well as understanding all the possible microscopic variables in real systems such as disorder or a moiré superlattice which may alter the balance of anisotropies and change the ground state. To this end, studies that combine microscopic and macroscopic measurements on the same device hold an advantage. Our measurements shown in Fig. 3 confirm the lifting of the fourfold degeneracy of zLL with the energy separation of the $\nu =0$ state much larger than $\nu =\pm1$, and all of them much larger than the bare Zeeman energy indicating interaction-dominated physics.

Theory and transport experiments have mainly converged that the CAF state is the ground state of the zLL in graphene at $\nu =0$, although more recent analysis suggests that the graphene ground-state physics is a complex and unsettled issue^29,48^. The convergence to the CAF state stems from the prediction that the excitation gap closes in a tilted magnetic field^28^, as shown schematically in Fig. 6e, and supported by transport measurements of a metal–insulator transition as a function of tilt angle of magnetic field^26^. However, for pure perpendicular magnetic fields, the valley isospin anisotropy is much larger than the Zeeman energy leading to the CAF state approaching the AF ground state (Fig. 6d)^26,28,49^. In fact, the CAF state bridges smoothly between the AF and F states as the angle between sublattice spin polarizations varies from $\pi /2$ to 0, respectively (see Fig. 6d–f)^28^. In this regard, the measurements in Fig. 6a are consistent with a CAF ground state.

Figure 6b–f shows possible edge spatial dispersions for other bulk phases including the KD and CDW phases. Both the KD and CDW phases show gapped excitations but now with all four levels separated in the bulk and dispersing to the edge (Fig. 6b, c). Both these phases have dispersions consistent with the results in Fig. 6a, in particular the fourfold splitting in the bulk, and show the best agreement with the experiment in Fig. 6a. Evidence for a KD phase has been observed in recent STM LDOS measurements at zero density^50^. Note that the ground-state phases are likely density-dependent, and even more complex behavior is possible as a bulk ground state can change its order parameter at the boundary as shown in recent calculations^29^.

In summary, we have obtained spatial measurements of the dispersion of the broken-symmetry edge channels near the quantum Hall boundary. The measurements alone cannot identify the ground-state symmetry in this particular sample but are consistent with edge profiles of various bulk phases as discussed above. Further measurements are required that can shed light on the ground-state properties such as atomically resolved measurements of the QH wavefunction symmetry as a function of distance near the QH boundary. Finally, we point out the unique benefits of KPFM as a new tool for Landau level spectroscopy which complements scanning tunneling spectroscopy. In addition, we demonstrated the combination of macroscopic transport and microscopic scanning probe measurements which advantageously removes uncertainties for a direct comparison of different techniques ensuring that all microscopic variables are key for defining the physics, such as disorder or moiré superlattices, are exactly the same in both measurement modalities.

## Methods

### Graphene device structure and fabrication

Figure 1a, b shows a schematic cross-section of the graphene device heterostructure and an optical top view. Two single-crystal graphite gate electrodes and single-crystal hBN dielectrics are employed for optimal sample quality. The two graphite back gate regions G1 and G2 are outlined in Fig. 1b. G1 defines the carrier density of the local interior area as indicated by the red dashed line in Fig. 1b, while G2 defines the carrier density in the outer region (blue dashed line). A quantum Hall boundary edge can thus be generated at the edge of the local gate G1.

The heterostructure is assembled from top to bottom (starting from the global graphite gate G2 as the top layer) using the van der Waals transfer technique so that the bottom of the graphene flake, as well as the hBN dielectrics, remain free from contamination during the stacking and subsequent fabrication processes. It is then flipped upside down to expose the graphene surface and deposited onto a 285 nm SiO_2_/ Si^++^ substrate before vacuum annealing to remove the polymer film underneath the stack. Electrical connections to the graphene sheet and graphite gate electrodes were made by deposition of the Cr/Pd/Au (2/50/50 nm) metal edge contacts.

All but one electrode contacting the graphene are in contact with both the G1 region and the G2 region. The one outside contact is used to ensure that the G2 region is in the $\nu =0$ gapped state during electrical transport and scanning probe measurements. A fan-shaped pattern with gold ridges of 65 nm height is connected to the drain electrode to the graphene sheet for navigation purposes (Fig. 1b). After introducing the device sample to the UHV chamber of the scanning probe microscope (SPM) instrument, it is annealed at a temperature of ≈623 K for ≈3 h to obtain the required cleanliness for SPM measurements.

### Navigating to the device with a scanning probe microscope

Navigating to the central device area with scanning probes is always a difficult challenge. For this purpose, a fan-shaped pattern extending to ≈500 µm at its widest region is utilized. Supplementary Fig. 1 outlines the procedure for navigating to the device area. First, the probe tip is aligned onto the fan-shaped area using an optical telescope while the STM module is in the upper ultrahigh vacuum chamber at room temperature (Supplementary Fig. 1a). Using the probe tip reflection, a tip-sample gap of the order of 100 µm is set at room temperature. The module is then transferred and locked into the dilution refrigerator multimode SPM system where it is cooled to a temperature of 10 mK^37,38^. The landing region is scanned, and a ridge is identified after approaching the fan-shaped runway surface. STM tunneling current or AFM frequency shift feedback can be used for the approach; for this device, AFM feedback was used for approach and navigation to minimize the degradation of the probe tip due to interacting with the surface. Once a ridge on the fan-shaped area is found, an automated algorithm is used to follow a given ridge to the device area (Supplementary Fig. 1b). This algorithm alternates stepping along the ridge direction and quickly scanning the ridge in a “W”-shaped line. After each “W” scan, the *XY* piezo motor parameters are adjusted to keep the walking direction along the ridge. The successful application of this routine is shown in (Supplementary Fig. 1c), where AFM traces of the “W” line scans are shown. At certain key places, full AFM scans are made to verify marker features in the devices, as shown in (Supplementary Fig. 1c). After successful navigation, the device region is located as verified by AFM scans of the pattern boundary (Supplementary Fig. 1d). Further navigation is then performed to check the device area and locate an area for edge studies, as indicated by the black circle in Fig. 2a.

### Multimode STM, AFM, and transport instrumentation

The study described in this report is the first to use a newly commissioned multimode system with the capabilities of simultaneous AFM, STM, and magnetotransport measurement^38^. The system utilizes a dilution refrigerator which operates at a base temperature of 10 mK with magnetic fields up to 15 T perpendicular to the sample plane^37^. Multimode measurements are accomplished by using custom-designed sample and probe tip holders which feature eight electrical contacts for devices and probe sensors. Magnetotransport measurements were performed using a lock-in amplifier at 25 Hz with a 10 nA source current. The qPlus AFM sensor was a new design that incorporated an integrated excitation electrode on the sensor^38^. The qPlus sensor had a quality factor of $Q=1.3\times {10}^{5}$ and a resonance frequency of *f*_0_ = 23.4 kHz at zero magnetic field. For the qPlus sensor, four contacts were wired, two to read out the AFM sensor, one for the STM tunneling current, and one for the sensor excitation. All eight electrical contacts of the device were utilized. For AFM and KPFM measurements, we used the frequency modulation mode with an oscillation amplitude of 2–5 nm. For KPFM, we used 1 Hz to 5 Hz modulation on the sample bias voltage.

### AFM frequency shift measurement of broken-symmetry states

AFM measurements of the frequency shift and dissipation were both sensitive to the occurrence of the broken-symmetry states, and Landau levels in general, as shown in Fig. 2. The sensitivity originates from the frequency shift caused by the capacitive forces due to unbalanced electrostatic potentials between the tip and the sample:

2 $$\triangle {f}_{C} \sim -\frac{{d}^{2}C\left({f}_{0}\right)}{d{z}^{2}}{\left({V}_{{\rm{B}}}-{V}_{{\rm{CPD}}}\right)}^{2}$$

where $C\left({f}_{0}\right)$ is the capacitance between the tip and the sample at the sensor resonance frequency ${f}_{0}$, ${V}_{{\rm{CPD}}}$ is the contact potential difference and ${V}_{{\rm{B}}}$ is the sample bias. The typical frequency shift curves are shown in (Supplementary Fig. 3d). One can see, for example, that at ${V}_{{\rm{B}}}-{V}_{{\rm{CPD}}}\cong 0.5$ V along the horizontal axis in (Supplementary Fig. 3d), the frequency shift is $\approx -0.15$ Hz.

The additional positive frequency shift at integer filling factors corresponding to the broken symmetry states seen in (Supplementary Fig. 2c, d) derives from the openings of gaps which change the resistance and, as a result, the capacitance $C\left({f}_{0}\right)$ of the graphene system at the tip location.

An additional dissipation develops once the complex capacitance $C\left({f}_{0}\right)$ acquires a phase lag resulting from a large local resistance of the incompressible region. As the resistance grows further, the capacitance $C\left({f}_{0}\right)$ decreases, correspondingly causing a positive frequency shift contribution, while the phase lag, and respectively the additional dissipation vanishes. The experimentally observed positive frequency spikes at the integers can be as large as 10–15% of the total frequency shift from the Coulomb attraction (≈0.02 Hz/0.15 Hz).

In the simplified analysis above, all the changes in $\triangle {f}_{C}$ were assigned to the changes in $C\left({f}_{0}\right)$ assuming ${V}_{{\rm{CPD}}}$ and ${V}_{{\rm{B}}}$ being constant. (Supplementary Fig. 2a) illustrates that over a larger parameter range, the latter two variables contribute most significantly. The frequency shift at the broken-symmetry gaps is a small signal on top of a large background due to the larger frequency shifts caused by changes in ${V}_{{\rm{CPD}}}$ originating from the normal cyclotron gaps at filling factors of $\nu =\pm2$ (along the gate voltage axis), as well as by ${V}_{{\rm{B}}}$ (the sample bias axis), both determining the total electrostatic force contributions. We followed refs. ^51,52^ and subtracted a smoothly varying background (black curve) from each frequency shift curve (red and blue curves) and plot the residuals, as shown in (Supplementary Fig. 2b, c). The smooth background averages the original data using a Gaussian filter with a sigma of 0.2 V. Each residual curve is then built into a new frequency shift map, as shown in (Supplementary Fig. 2d) and Fig. 2e.

As mentioned above, the frequency shift data contains contributions from electrostatic forces which give rise to a downward parabolic dependence on the applied sample bias (Supplementary Fig. 3d). The vertex of the parabolic response occurs when the applied potential compensates the contact potential difference (CPD) between the probe and graphene, as illustrated in (Supplementary Fig. 3a–c). By measuring changes in the CPD, we can obtain a measure of the local chemical potential, which responds to changes in Fermi-level position with gate bias, as shown in (Supplementary Fig. 3e). The measurements of the chemical potential in (Supplementary Fig. 3e) were obtained by fitting the parabolic dependence on the sample bias over a window of 300 mV about the vertex. A higher-precision measurement with reduced tip-gating effects is obtained by modulating the sample bias and using lock-in detection to measure the sample bias values compensating the CPD. The force at the modulation frequency ω is:

3 $${F}_{\omega }=-\frac{1}{2}\frac{{{\mathrm{d}}C}}{{{\mathrm{d}}z}}{\left({V}_{{\rm{B}}+\text{AC}}-{V}_{{\rm{CPD}}}\right)}^{2} \sim -\frac{{{\mathrm{d}}C}}{{{\mathrm{d}}z}}\left({V}_{\text{B}}-{V}_{\text{CPD}}\right){V}_{\text{AC}}$$

Correspondingly, $\triangle {f}_{\omega } \sim \frac{{\mathrm{d}}{F}_{\omega }}{{{\mathrm{d}}z}}$ is nullified when ${V}_{{\rm{B}}}-{V}_{{\rm{CPD}}}=0.$ This method was used in the Kelvin probe measurements in Figs. 3–6.

All AFM measurements were made using a constant amplitude signal where the dissipation is measured in the excitation drive signal required to keep the amplitude constant with the exception of the data in Fig. 2e, f (and Supplementary Fig. 2) where a constant excitation signal was used and the dissipation was measured in the amplitude signal channel.

### KPFM chemical potential excursions in the *N* = 0 Landau level

In the *N* = 0 Landau level, an “N”-shaped excursion in the chemical potential is observed at the integer filling factors between transitions of the mini-plateaus that make up the *N* = 0 LL. The origin of this excursion is unclear, as it has the opposite sign to previous measurements of the compressibility at the zeroth LL^42^ using single-electron transistors, and experimental artifacts need to be considered. However, the feature is quite robust and, at present, we cannot identify it as an artifact based on the following arguments: (1) the excursion appears both in the KPFM chemical potential and the AFM frequency shift and dissipation signals, without and with local tip gating, (2) the feature is seen for wide and narrow strips in different spatial locations (see Fig. 5), indicating that local resistive effects are likely not contributing, and (3) the feature is seen near local integer filling factors (see Fig. 5), indicating that non-local resistive/capacitive effects that can be caused by large (bulk) areas of the sample becoming insulating at integer filling factors are not contributing. In addition, the following arguments against a spurious origin of the signal as: (i) the feature is odd with respect to gate voltage, an artifact due to increased resistance for example would be symmetric about the integer fillings, and (ii) the feature was independent of the direction of gate voltage sweeps.

## Supplementary information

Supplementary Information

## Data Availability

All data are available in the main text or the Supplementary Materials and are available from the corresponding authors upon reasonable request.
