# Supplementary material for: Edge channels of broken-symmetry quantum Hall states in graphene visualized by atomic force microscopy
Source: Nat Commun. 2021 May 14;12:2852. doi: 10.1038/s41467-021-22886-7 (PMC8121811; doi:10.1038/s41467-021-22886-7)
Supplement: Supplementary file 1 — Supplementary Information [file 41467_2021_22886_MOESM1_ESM.pdf]

Supplementary Information for

Edge Channels of Broken-Symmetry Quantum Hall States in Graphene visualized by Atomic Force Microscopy

Sungmin Kim[1,2*], Johannes Schwenk[1,2*], Daniel Walkup[1,2*], Yihang Zeng[3*], Fereshte Ghahari[1,2], Son T. Le[1,4], Marlou R. Slot[1,5], Julian Berwanger[6], Steven R. Blankenship[1], Kenji Watanabe[7], Takashi Taniguchi[8], Franz J. Giessibl[6], Nikolai B. Zhitenev[1], Cory R. Dean[3†], and Joseph A. Stroscio[1†]

[1]Physical Measurement Laboratory, National Institute of Standards and Technology, Gaithersburg, MD 20899, USA.
[2]Institute for Research in Electronics and Applied Physics, University of Maryland, College Park, MD 20742, USA.
[3]Department of Physics, Columbia University, New York, NY 10027, USA.
[4]Theiss Research, La Jolla, CA 92037, USA.
[5]Department of Physics, Georgetown University, Washington, DC 20007, USA.
[6]Institute of Experimental and Applied Physics, University of Regensburg, Regensburg D-93040, Germany.
[7] Research Center for Functional Materials, National Institute for Materials Science, Tsukuba, Ibaraki 305-0044, Japan.
[8]International Center for Materials Nanoarchitectonics, National Institute for Materials Science, Tsukuba, Ibaraki 305-0044, Japan

†Correspondence to: cdean@phys.columbia.edu, joseph.stroscio@nist.gov

[*] These authors contributed equally to this work.

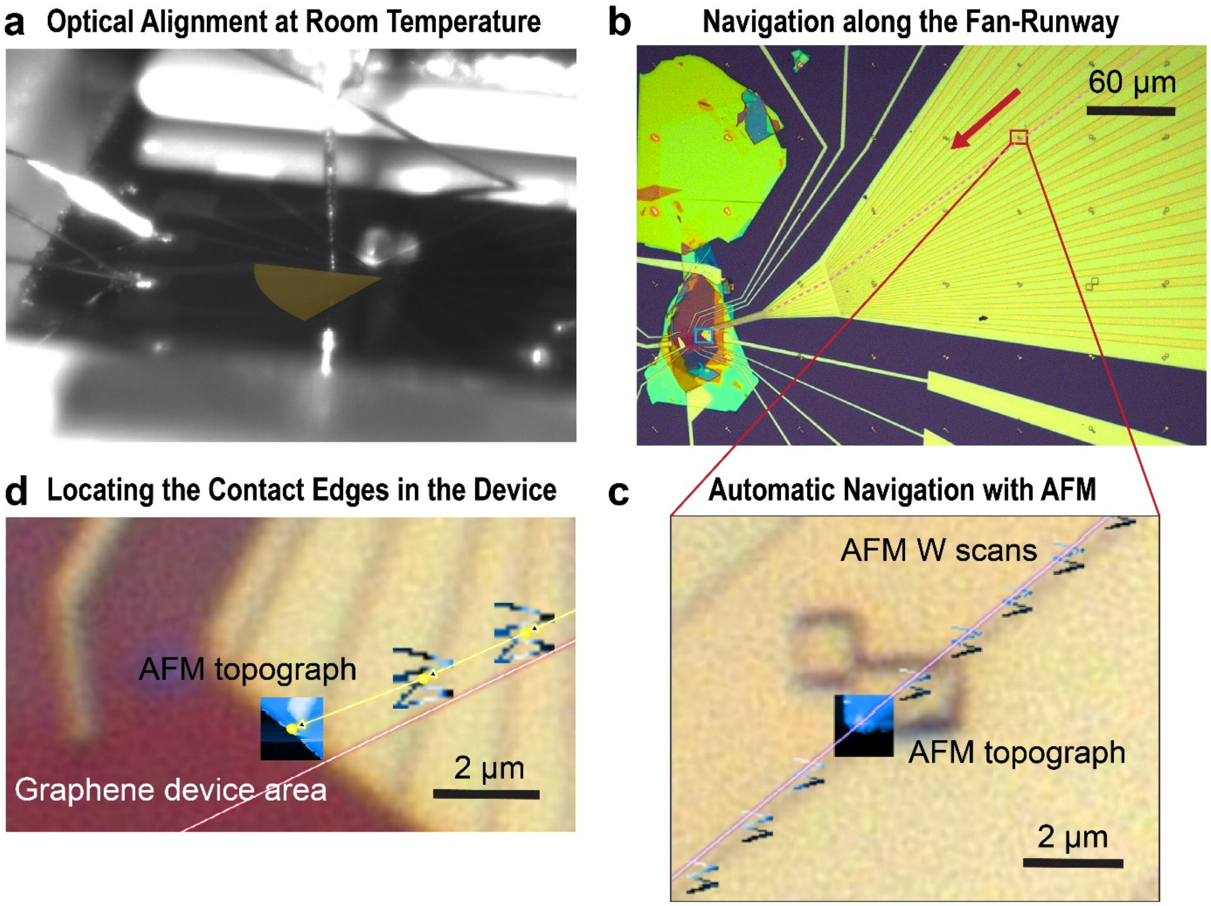

Supplementary fig. 1 | SPM navigation to central device area. **a,** Optical image of the probe tip and its reflection as it is aligned onto the graphene device. The fan-shaped runway is artificially highlighted in yellow. **b,** Optical image of the fan-shaped runway and device area (on the left). The red arrow indicates the navigation direction and pink dashed line shows the navigating pathway. **c,** Zoomed-in optical image of a part of the navigation path. An AFM image and AFM "W"-shaped scans along the navigation path are superimposed. As shown, the automated algorithm repeats "W"-shaped AFM linescans to sense the ridge edge and re-calculate the next X and Y piezo-motor steps to adjust the direction. Along the navigation path, several full-frame scans are taken as shown to reveal markers to ensure accurate alignment of the navigation system to the optical image. **d,** Optical image of the contact edge of the runway displaying the final navigation to the graphene device area. The AFM image showing the contact-graphene area is superimposed on the optical image. AFM topographs in (**c**) and (**d**) are measured at 2 nm oscillation amplitude and set frequency shift of −30 mHz.

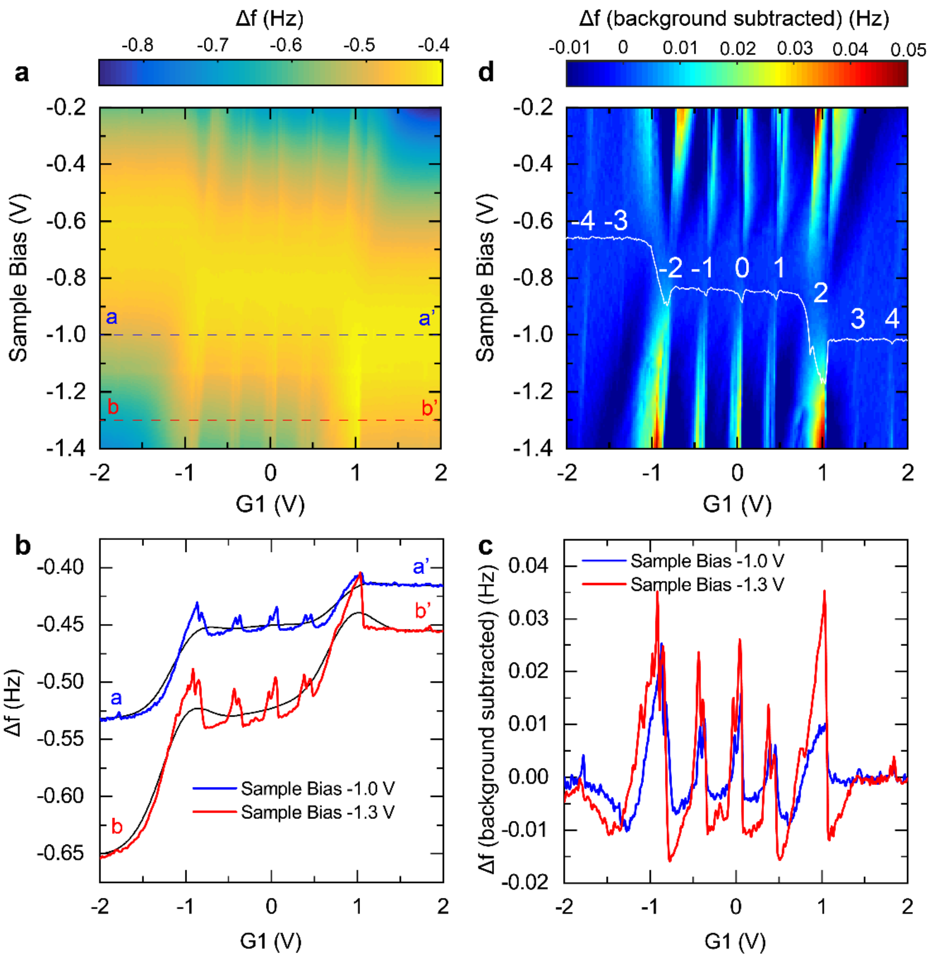

Supplementary fig. 2 | AFM frequency shift measurement of broken-symmetry states. a, Raw frequency shift data as a function of sample bias and local back gate G1; the global back gate G2 is set to zero density. The frequency shift changes due to broken-symmetry states, seen as sharp spikes in the middle of the image, are superimposed on a pronounced background. The background is dominated by large steps corresponding to chemical potential changes at filling factors of $\nu = \pm 2$ along the gate axis, and electrostatic forces along the sample bias axis. AFM data is measured at $B = 15$ T, with a constant excitation of 520 mV. The blue and red dashed lines indicate the positions for the linescans in (b). **b,** Linescans from (a) along the gate axis at **a-a'** and **b-b'**, corresponding to sample biases of $V_\mathrm{B} = -1.0$ V, and -1.3 V, respectively. The solid back line is a smooth background calculated for each linescan using a Gaussian filter with $\sigma = 0.2$ V. **c,** Residuals obtained from subtracting the background curves shown in (**b**) from the raw data. **d,** Frequency shift map obtained from the background-corrected linescans as in (**c**) for each sample bias, to highlight the changes due to the broken-symmetry states. The white line reflects the gate-dependent zero-contact potential, as obtained following the procedure in (Supplementary Fig. 3). The white numerals indicate the filling factor.

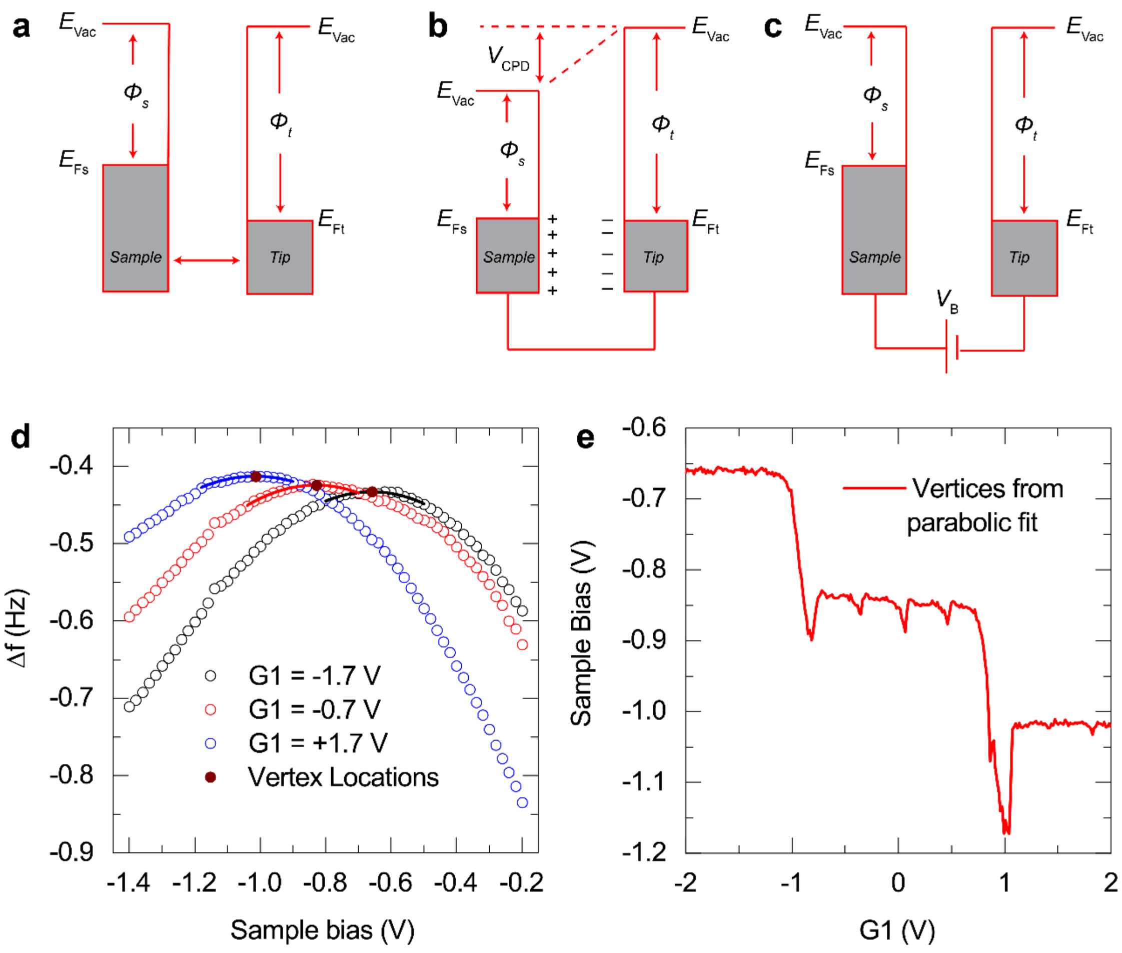

Supplementary fig. 3 | Kelvin probe force spectroscopy of Landau levels. Electronic energy levels of the probe tip and sample showing the Fermi level, E_{Ft}, E_{Fs}, vacuum level, E_{Vac}, and tip and sample workfunctions, φ_{t}, φ_{s}, for three cases: **a,** Tip and sample are disconnected and separated by a large distance. **b,** Tip and sample are in electrical contact resulting in charge flow and alignment of Fermi levels. A contact potential difference (CPD) is built up resulting in an electric field between the sample and tip. **c,** A sample bias is applied between the sample and tip to null the CPD. **d,** Linescans along the sample bias axis from (Supplementary Fig. 2a) at selected gate voltages showing a downward parabolic response (symbols). The vertex of each parabola is obtained by fitting the data over a window of 0.3 V about the vertex (solid lines). The shift in the vertex positions is due to changes in chemical potential with gate voltage G1. **e,** Vertex positions from fitting the downward parabolic response as in (D) for each gate voltage. The large steps are due to Landau levels at filling factors of $\nu = \pm 2$, while smaller steps are seen inside the $N = 0$ Landau level due to broken-symmetry states. Note that the chemical potential has a sign change along the sample bias axis as shown in Fig. 3 of the main text, where the KPFM data is obtained with lock-in measurements for improved signal to noise.

Supplementary note 1: Spatial Mapping of Edge Channels with AFM Dissipation Measurements

The dispersion of the edge channels can also be mapped using the dissipation signal in the AFM measurement, which gives more contrast to the edge channel features. The dissipation signal is the excitation voltage needed to keep a constant oscillation amplitude of 2 nm of the AFM qPlus sensor. (Supplementary Fig. 4) shows a series of 400 nm by 400 nm spatial images of the AFM dissipation signal over the electrostatically defined boundary of the device. The images resolve the $\nu = \pm 1$ edge states at B = 10 T at various local-gate potentials across the QH boundary, indicated in the black circular region in Fig. 2a of the main text. The first row of images shows the incompressible strip corresponding to $\nu = -1$ appearing in the local gate voltage range of -280 mV $<$ G1 < -100 mV. The $\nu = -1$ strip is detected near the boundary at lower potentials and moves up in the images away from the boundary with increasing gate potential (density) as the central region of the Hall bar over the local gate approaches $\nu = -1$, as indicated by the white arrow. The second row in (Supplementary Fig. 4) shows the emergence of the $\nu = 0$ state occurring inside the boundary and merging with the outside $\nu = 0$ region at G1 $= 110$ mV. Finally, the third row of images shows the electron counterpart of the top row with the $\nu = +1$ incompressible state, first appearing from the top of the image and then drifting toward the boundary edge with increasing gate potential (310 mV $<$ G1 $<$ 430 mV). To relate the images to the dispersion in Fig. 5b of the main text, locate the gate voltage along the horizontal axes, and then the image in (Supplementary Fig. 4) would correspond to the data along the horizontal line in Fig. 5b, but now scanned along the X-axis in the dissipation channel. The dispersion of broken-symmetry state $\nu = -1$ dispersing to below $\nu = 0$ in Fig. 5a-b is seen as the dissipative channel shifting down with decreasing gate voltage in the first row of images

(note the Y-axis is horizontal in Fig. 5). Similarly, the dispersion of the $\nu = +1$ to above $\nu = 0$ in Fig. 5 is seen as the dissipative channel shifting up with increasing gate voltage in the third row of images in (Supplementary Fig. 4). Overall, the XY-maps in (Supplementary Fig. 4) show the dispersion identical to Fig. 5a-b, progressing uniformly along the QH boundary edge. Since the dissipation measurements are operated off balance, a finite tip gating occurs and filamentary arcs are observed similar to reported in scanning capacitance measurements of AlGaAs/GaAs 2DEG [1].

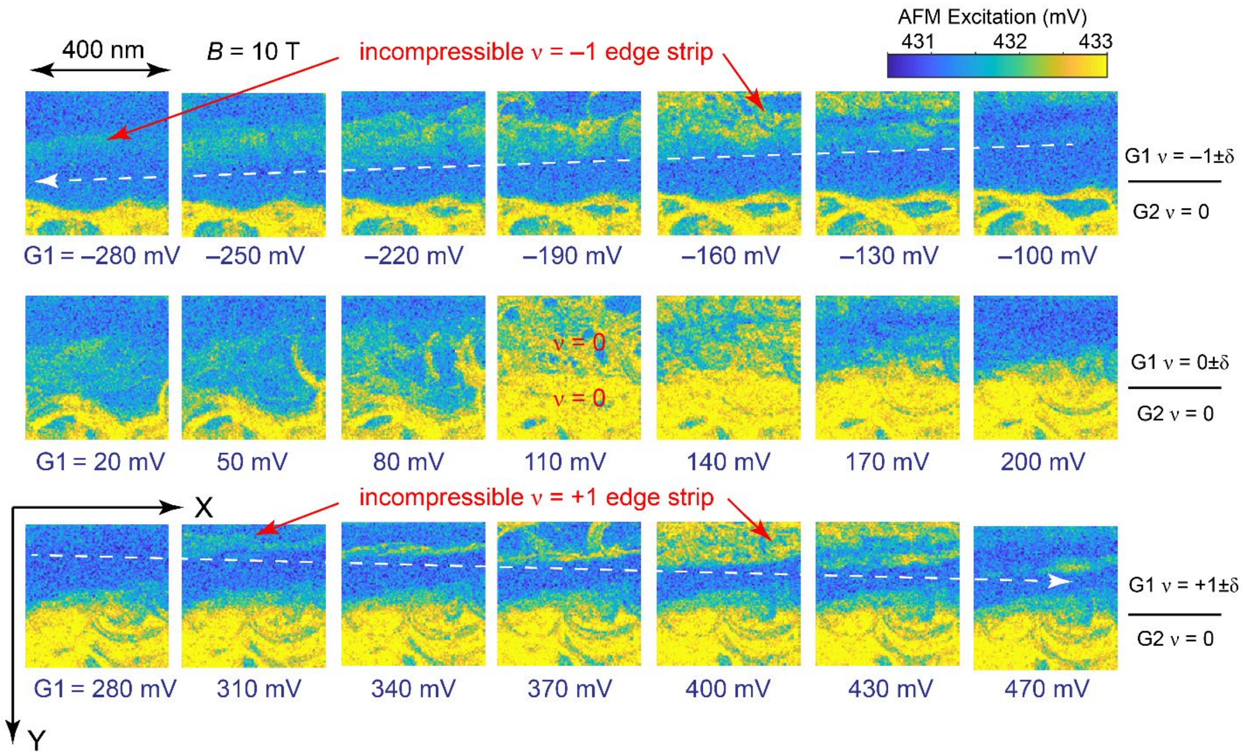

Supplementary fig. S4 | Spatial mapping of graphene broken-symmetry quantum Hall edge states using AFM dissipation measurements. Series of spatial AFM excitation channel maps (for constant sensor oscillation amplitude of 2 nm) across the QH edge boundary (indicated in the black circle in Fig. 2a) as a function of the local back gate potential G1. The gate potential outside the boundary was set to $\nu = 0$, an electronic insulator. (first row) The incompressible $\nu = -1$ edge state appears out of the boundary and drifts up with increasing gate voltage. (second row) The $\nu = 0$ appears at the local gate voltage of $\text{G1} = 110$ mV. (third row) The incompressible $\nu = +1$ edge state appears from the top of the frame and drifts down with increasing gate voltage. AFM settings: $V_B = -600$ mV, $\Delta f = -2$ Hz.

Supplementary References:

1. Steele, G. A., Ashoori, R. C., Pfeiffer, L. N. & West, K. W. Imaging Transport Resonances in the Quantum Hall Effect. *Phys. Rev. Lett.* **95**, 136804 (2005).
